# Supplementary material for: Effects of Low-Salinity Stress on Histology and Metabolomics in the Intestine of Fenneropenaeus chinensis
Source: Animals (Basel). 2024 Jun 26;14(13):1880. doi: 10.3390/ani14131880 (PMC11240639; doi:10.3390/ani14131880)
Supplement: Supplementary file 1 [file animals-14-01880-s001.zip › animals-3036341-supplementary.pdf]

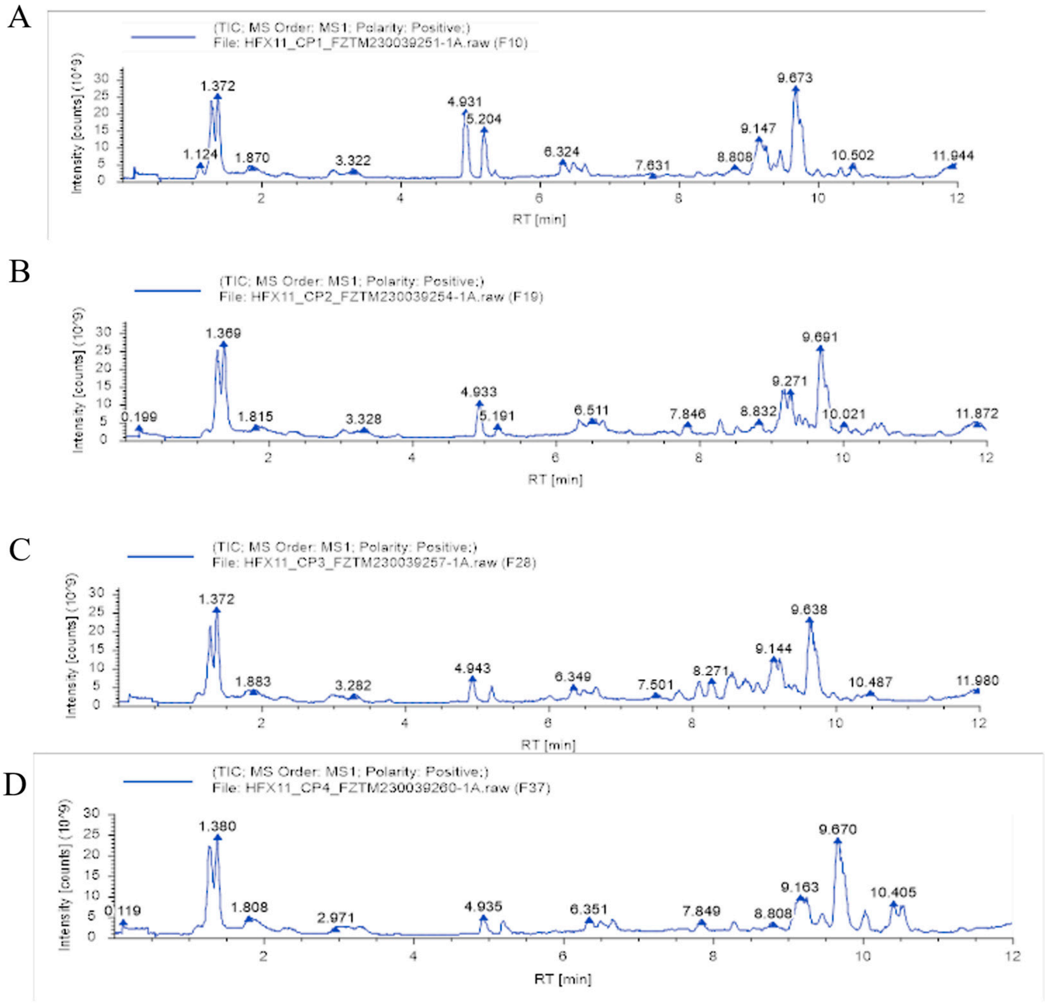

Figure S1. Representative liquid chromatography–mass spectrometry (LC–MS) total ion chromatograms (TICs) of quality control (QC) in *F. chinensis*. (A) C0, (B) S3, (C) S7, and (D) S14.

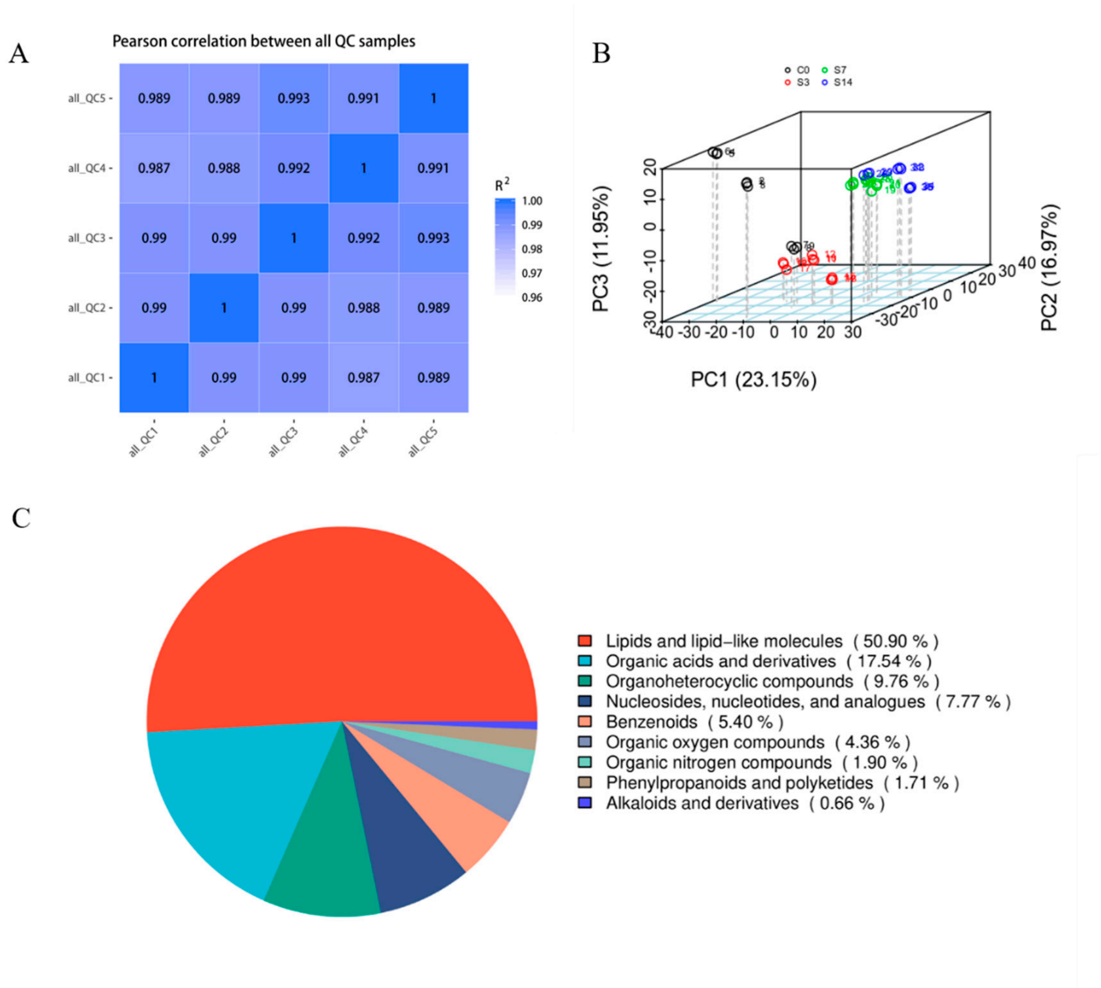

Figure S2. Information of between all QC sample and initial data analysis. (A) pearson correlation. (B) three-dimensional spatial PCA analysis and (C) metabolic signals were categoried.

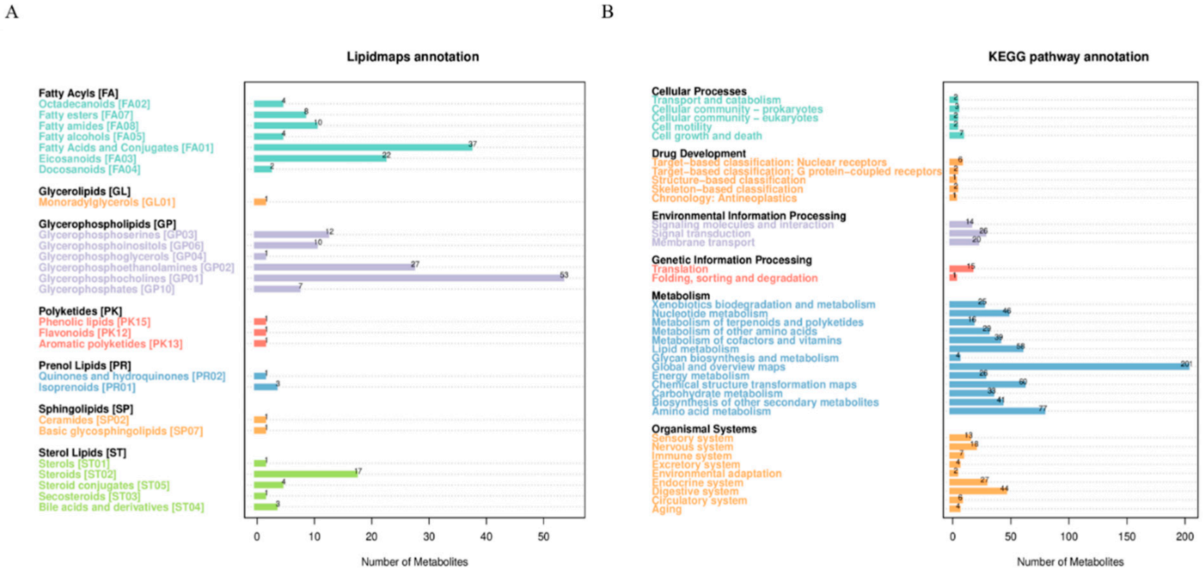

Figure S3. Annotation of intestine metabolites in *F. chinensis* under low-salinity stress. (A) Lipidmaps and (B) KEGG pathway was annotated. The X-axis represents the number of metabolites. The Y-axis represents the annotated Lipidmaps or KEGG terms.

Table S1. Metabolite differential screening results.

	Compared Samples	Num. of Total Ident.	Num. of Total Sig.	Num. of Sig. Up	Num. of Sig. down	Num. of Sig. POS/ NEG
POS	S3	1050	280	158	122	1024
	S7	1050	366	178	188	
	S14	1050	378	178	200	
NEG	S3	409	102	25	77	394
	S7	409	124	35	89	
	S14	409	168	96	72	

Table S2. Significant differential metabolic pathways in the intestine of *F. chinensis* in response to low-salinity stress.

Groups	MapID	MapTitle	Pvalue	MetaIDs
S3	map00400	Phenylalanine, tyrosine and tryptophan biosynthesis	0.002604	Indole; D-Erythrose 4-phosphate; L-Phenylalanine; D-Fructose 1,6-bisphosphate; Tryptophan; Quinic acid; Tyrosine; Phenylpyruvic acid
	map04917	Prolactin signaling pathway	0.003554	Levodopa; Estradiol; L-Dopa; Progesterone; D-Glucose 6-phosphate
	map00052	Galactose metabolism	0.034695	D-Fructose 6-phosphate; Inositol; Sucrose
	map00562	Inositol phosphate metabolism	0.034695	Inositol; D-myo-Inositol 1,4-bisphosphate; D-Glucose 6-phosphate
	map04973	Carbohydrate digestion and absorption	0.034695	Maltotriose; Sucrose; D-Glucose 6-phosphate
	map05012	Parkinson's disease	0.034695	Levodopa; Adenosine; L-Dopa
	map05034	Alcoholism	0.034695	Levodopa; Adenosine; L-Dopa
S7	map02010	ABC transporters	0.018652165	Biotin; Maltotriose; Choline; Inositol; Sucrose; Betaine; Glutathione; L-Phenylalanine; L-Threonine
S14	map00061	Fatty acid biosynthesis	0.004678984	Stearic Acid; Lauric Acid; Oleic Acid; Palmitoleic Acid; Palmitic Acid; Myristic Acid
	map00360	Phenylalanine metabolism	0.017682002	Phenylglyoxylic acid; 4-Hydroxybenzoic acid; Hippuric acid; Phenylacetylglycine; Succinic acid; L-Phenylalanine; Vanillin; D-Phenylalanine; L-Tyrosine; Phenylpyruvic acid

Table S3. Results of KEGG significant enrichment analysis of 14 overlapping DEMs.

DMs	MetaIDs	MapID	MapTitle	Pvalue
Down	Hippuric acid<br>Phenylpyruvic acid D-Phenylalanine L-Phenylalanine<br>Succinic acid<br>Phenylacetylglycine	map00360	Phenylalanine metabolism	0.000223247
	Phenylpyruvic acid<br>Succinic acid<br>L-Phenylalanine<br>Tryptophan	map00400	Phenylalanine, tyrosine and tryptophan biosynthesis	0.006050961
	6-Hydroxymelatonin<br>Succinic acid<br>L-Kynurenine<br>Tryptophan	map00380	Tryptophan metabolism	0.012691005
	L-Phenylalanine<br>Maltotriose<br>Sucrose	map02010	ABC transporters	0.022832686

	Biotin<br>Phenylpyruvic acid<br>L-Phenylalanine<br>Tryptophan	map01210	2-Oxocarboxylic acid metabolism	0.044154429
	Vitamin A	map00830	Retinol metabolism	0.008163265
Up	L-Palmitoylcarnitine	map00071	Fatty acid degradation	0.016293075
	L-Palmitoylcarnitine	map01212	Fatty acid metabolism	0.016293075
